# Can self-testing increase HIV testing among men who have sex with men: A systematic review and meta-analysis

**DOI:** 10.1371/journal.pone.0188890

**Published:** 2017-11-30

**Authors:** Ci Zhang, Xianhong Li, Mary-Lynn Brecht, Deborah Koniak-Griffin

**Affiliations:** 1 Xiangya Nursing School of Central South University, Changsha, Hunan, China; 2 UCLA School of Nursing, Los Angeles, California, United States of America; National and Kapodistrian University of Athens, GREECE

## Abstract

**Background:**

Globally, four out of ten individuals living with HIV have not been tested for HIV. Testing is especially important for men who have sex with men (MSM), among whom an increasing HIV epidemic has been identified in many regions of the world. As a supplement to site-based HIV testing services, HIV self-testing (HIVST) provides a promising approach to promote HIV testing. However, evidence is scattered and not well-summarized on the effect of HIVST to improve HIV testing behaviors, especially for MSM.

**Methods:**

Seven databases (PubMed, Web of Science, Cochrane Library, PsycINFO, CINAHL Plus, WanFang, and China National Knowledge Internet) and conference abstracts from six HIV/sexually transmitted infections conferences were searched from January 2000-April 2017.

**Results:**

Of 1,694 records retrieved, 23 studies were identified, 9 conducted in resource-limited countries and 14 in high-income countries. The pooled results showed that HIVST increased HIV test frequency for MSM by one additional test in a 6-month period (mean difference = 0.88 [95% CI 0.52–1.24]). The pooled proportion of first-time testers among those who took HIVST was 18.7% (95% CI: 9.9–32.4) globally, with a rate 3.32 times higher in resource-limited country settings (32.9% [95% CI: 21.3–47.6]) than in high-income countries (9.9% [95% CI: 7.4–13.8]). The pooled proportions included non-recent testers, 32.9% (95% CI: 28.1–38.3); ever or currently married MSM, 16.7% (95% CI: 14.5–19.4); and HIV positive men, 3.8% (95% CI: 2.0–5.7) globally; 6.5% [95% CI: 0.38–12.3] in resource-limited country settings; and 2.9% [95% CI: 2.0–5.0] in high-income countries). The rates reported for linkage to care ranged from 31.3% to 100%.

**Conclusions:**

HIVST could increase HIV testing frequency and potentially have capacity equivalent to that of site-based HIV testing services to reach first-time, delayed, married, and HIV-infected testers among MSM and link them to medical care. However, more rigorous study designs are needed to explore the specific self-testing approach (oral-fluid based or finger-prick based) on improving HIV testing for MSM in different social and economic settings.

## Introduction

Globally, approximately 36.7 million individuals are living with HIV, with about 2.2 million new cases identified annually over the past 5 years[1–4]. Notably, men who have sex with men (MSM) account for 30% of all HIV infected people in the world [3], and 49% of the cases in MSM are reported in Western Europe, Central Europe, and North America [3]. The HIV epidemic is rapidly growing among MSM in some regions of the world, especially in China with 29.4% of new infections occurring among MSM [5]. However, an estimated 4 in 10 of HIV positive individuals, globally, have not been tested for HIV [4]. A recent meta-analysis synthesizing studies from China showed that about half of Chinese MSM had never been tested for HIV in their lifetime, and 62% had not been tested in the past 12 months [6]. Inadequate uptake of HIV testing, high frequency of unprotected sexual behaviors, and multiple sexual partners may potentially fuel the HIV epidemic among the MSM population, and may also bridge the epidemic to the general population through heterosexual contact with women, especially in developing and undeveloped countries [7,8]. Late testing and late access to treatment also remain a main cause of HIV/AIDS-related deaths[9]. Therefore, improving HIV testing becomes the first and key step to achieve, by 2020, the "90-90-90" goal set up by UNAIDS in 2014 (90% of people with HIV get tested and diagnosed, 90% of those diagnosed get treatment, and 90% of individuals on treatment get virally suppressed)[10].

HIV self-testing (HIVST) provides a promising approach to expand HIV testing. HIVST is defined as an approach in which individuals collect their own specimens and then conduct a test and read the results by themselves [11], thus, the dried blood spot (DBS) self-collection approach is excluded, because the test is not performed and the result is not read by the client themselves. There are two types of self- testing: one is oral-fluid based testing kits, and the other is finger- prick based kits. Currently only one oral HIV self-test kit is commercially available which was approved by the U.S. Food and Drug Administration (FDA) in 2012 (Ora Quick In-Home HIV Test). In most countries, oral self-testing kits are much more expensive than finger-prick self-testing kits [11]. The characteristics of more privacy, convenience, and confidentiality of HIVST were expected to facilitate increasing HIV testing, especially for MSM [12–14], but in reality, not as many MSM as predicted have used HIVST. For example, although HIVST kits are widely available and accessible in China, only 6.1% to 26.2% of MSM have ever used HIVST [15–17]. The barriers may include perceived unreliable results, no perception of HIV risk, concerns about correct HIVST operation, relatively high cost, fear of positive results, and concerns about lack of support if a test were positive[18]. Some experts have also worried about potential psychological risks and difficulty in linking positive individuals to treatment [19,20].

WHO (2016) recently released a new guideline on HIV self-testing and partner notification, with the recommendation to support trained lay providers to deliver HIVST using rapid diagnostic tests (RDTs) and emphasis on the need for strategic approaches to deliver the kits [11]. However, evidence is scattered and not well-summarized on the effect of HIVST to improve HIV testing for the specific group of MSM. This systematic review was conducted to address the knowledge gap by synthesizing the evidence on (1) whether HIVST could increase the HIV test frequency for MSM; (2) the capacity of HIVST to reach first-time testers among MSM; (3) the capacity of HIVST to reach non-recent testers; (4) the capacity of HIVST to reach the hidden subgroup of married MSM (with women); (5) the capacity of HIVST to identify more HIV-positive men; and (6) the linkage-to-care rate for HIV-positive men identified by HIVST (because of concerns that individuals using HIVST without supervision might fail to seek care following a positive result)[21].

## Methods

### Inclusion criteria

We conducted a systematic literature review to evaluate the current evidence on the effectiveness of HIVST to promote HIV testing among MSM. The target population was defined as MSM who reported ever taking HIVST. Studies were eligible if they reported data on at least one of the following outcomes: (1) the frequency of HIV testing; (2) the proportion of first-time testers; (3) the proportion of non-recent testers; (4) the proportion of ever or currently married MSM; (5) the proportion of people who had HIV positive results; (6) the proportion of linkage- to-care for those who had positive results through HIVST. The specific definitions of the six outcome variables are described in Table 1. Randomized controlled trials (RCTs), quasi-experimental studies, and observational (cross-sectional, case-control, and cohort) studies were eligible for inclusion in this meta-analysis. The intervention in RCT and quasi-experimental studies was restricted to HIVST only. Baseline data were used to conduct the meta-analysis for the above outcomes if the study was not observational. The study was excluded if it was not an original investigation, duplicated results from another study, or was qualitative.

**Table 1:** Definitions of the outcome variables.

Outcome variables	Definition
Frequency of HIV testing	The number of HIV tests that MSM took in the past 6 months (including all kinds of testing approaches)
First-time testers	Individuals who self-report having taken an HIV test for the first time
Non-recent testers	Individuals who self-report not having taken an HIV test in the past year
Ever or currently married MSM	MSM who were ever or are currently married to women
HIV-positive MSM	MSM who demonstrate HIV-positive results by using self-testing approaches
Linkage to care	MSM who demonstrate HIV-positive results using a self-testing approach, seek confirmatory testing in clinic/hospital/CDC, and get enrolled in a medical care or treatment program

This review was reported following PRISMA guidelines[22].

### Search strategy

**Fig 1:**
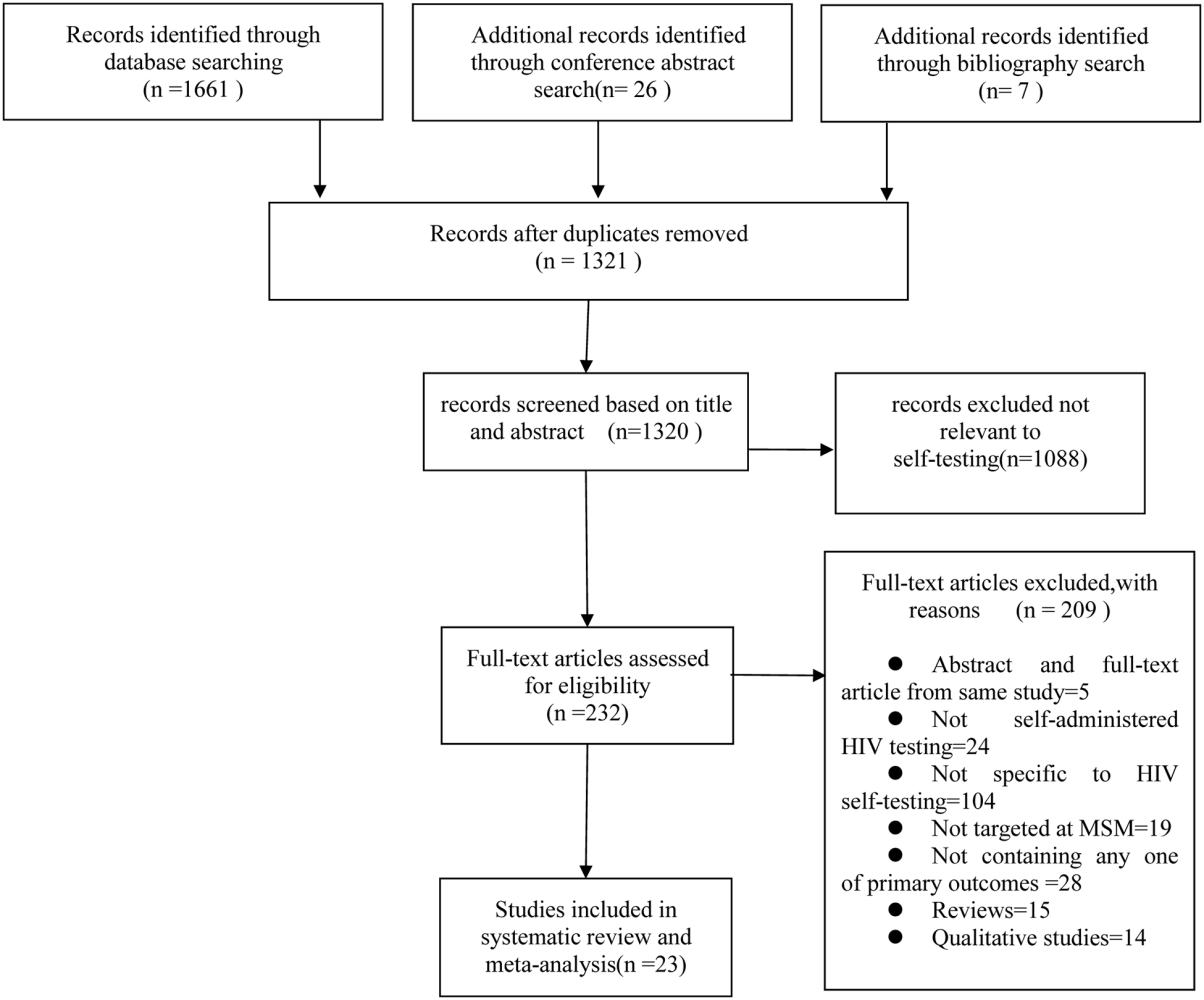
Flow chart of selection procedure and outcomes of this systematic review.

We searched 7 electronic databases (PubMed, Web of Science, Cochrane Library, PsycINFO, CINAHL Plus, WanFang, and the China National Knowledge Internet) and conference abstracts from International AIDS Society (IAS), International Congress of Behavioral Medicine (ICBM), Infectious Diseases Society of America (IDSA), Canadian Association of HIV Research (CAHR), HIV Diagnostics Conference (HDC) and International Society for Sexually Transmitted Diseases Research (ISSRTDR) for publications from January 2000 to April 2017. Our search terms (limited to humans) included (HIV OR HIV seropositivity OR HIV infection) AND ((self test*) OR (home*test*) OR (rapid*test*)) AND ((men who have sex with men) OR MSM OR homosexuality OR gay OR bisexual*)), using MeSH terms for PubMed and comparable terms for other databases. The search was limited to English-language and Chinese-language articles. There were no geographic limitations. Abstracts were included if full texts were not available. We screened gray literature through Google Scholar. Additionally, bibliographies of relevant papers were manually searched and the authors contacted for original data when needed. The details were listed in the flow chart (Fig 1).

### Data screening and extraction

Two reviewers (C.Z. and X.L.) independently screened titles and abstracts of the articles referring to MSM and HIVST. And then, full text and abstracts were screened for all eligibility criteria. Discrepancies (about 6%) were resolved by discussion after reviewing the full text. A standard data extraction form was used to extract variables from identified studies (Table 2).

**Table 2:** Characteristics of included studies.

No.	Author Year	Setting	Design	Sample Size	Product Kit	HIV Testing Frequency	The First Time Testers (%)	Non-recent Testers(%)	Married MSM(%)	HIV(+)Person (%)	Linkage to Care (%)	Score for Quality Critique (%)
1	Jamil 2017	Australia	Waiting-list RCT	Intervention:178;control:165	Oral-fluid	Intervention: mean 4.0 per year (95% CI: 3.7–4.3); control: mean 1.9 per year (95% CI: 1.7–2.2) RR 1.9 (95% CI:1.7–2.2)	NA	16.9% (30/178)	NA	1.7% (3/178)	100% (3/3)	100% (13/13)
2	Katz 2015	Seattle, USA	RCT	Intervention:115;control:115	Oral-fluid	Intervention:mean 5.3per15month(95% CI: 4.7,6.0); control:mean 3.6 per 15 month (95% CI: 3.2,4.0)	NA	NA	NA	NA	NA	46% (6/13)
3	Marlin 2014	Los Angeles, USA	QE	50	Oral-fluid	NA	NA	30% (15/50)	NA	6% (3/50)	100% (3/3)	67% (6/9)
4	Qin 2017	China	CS	341	Finger-prick 242/341 (71%); Oral-fluid 99/341 (29%)	79 (23%,341) reported HIV test frequency increased after first using HIVST	58.7% (200/341)	59% (117/143)	18% (117/647)	11.7% (40/341)	78% (31 /40)	100% (8/8)
5	Huang 2016	Los Angeles, USA	QE	122	Oral-fluid	NA	10.7% (13/122)	39% (47/122)	NA	1.6% (2/122)	100% (2/2)	67% (6/9)
6	Li 2016	Yunnan, China	QE	200	Oral-fluid	NA	NA	NA	21.5% (43/200)	23% (46/200)	NA	67% (6/9)
7	Wong 2015	Hong Kong	CS	68	Finger-prick (76.5%)	NA	NA	NA	NA	4.4% (3/68)	100% (3/3)	100% (8/8)
8	Greacen 2012	France	CS	69	NA	NA	12.2% (10/82)	50% (41/82)	NA	2.9% (2/69)	66.7% (2/3)	100% (8/8)
9	Zhong 2016	Guang Zhou, China	QE	198	NA	NA	27.8% (55/198)	45.5% (65/198)	8.1% (16/178)	4.5% (8/178)	100% (8/8)	56% (5/9)
10	Tao 2014	Beijing, China	QE	220	Finger-prick	NA	50.9% (112/220)	NA	20% (40/200)	15% (33/220)	100% (33/33)	56% (5/9)
11	Yan 2015	Jiangsu, China	CS	137	Finger-prick118 (86.1%);Oral-fluid 17 (13.9%)	NA	7.3% (10/137)	NA	17.5% (24/137)	NA	NA	100%(8/8)
12	Elliot 2017	London, UK	QE	5696	Oral-fluid or Finger-prick	NA	NA	NA	NA	1.6% (93/5696)	88% (82/93)	67% (6/9)
13	Woods 2016	San Francisco, USA	QE	53	Oral-fluid	NA	NA	20.8% (11/53)	NA	10% (5/53)	80% (4/5)	56% (5/9)
14	Zhou 2015	Beijing, China	QE	384	Oral-fluid	NA	NA	NA	NA	0.5% (2/384)	1.3% (5/16)	56% (5/9)
15	Grov 2016	America	QE	1071	Oral-fluid	NA	5.8% (62/1071)	38.5% (412/1071)	NA	0.9% (11/1268)	100% (11/11)	67% (6/9)
16	McDaid 2016	United Kingdom	CS	2409	Oral-fluid	NA	9.5% (230 /2409)	41.6% (1001/2409)	NA	3.8% (134/3549)	NA	88% (7/8
17	Daniels 2016	Los Angeles, USA	QE	37	NA	NA	NA	NA	NA	4% (1/37)	NA	67% (6/9)
18	Flowers 2017	Glasgow, Edinburgh, and Dundee, UK	MM	999	Oral-fluid	NA	15.4% (154/999)	NA	NA	5.3% (61/1151)	NA	88% (7/8)
19	Rosengren2016	Los Angeles, USA	QE	125	Oral-fluid	NA	8.8% (11/125)	37.6% (47/125)	NA	4% (2/56)	100% (2/2)	67% (6/9)
20	Carballo-Die′guez 2012	New York City, USA	QE	44	Oral-fluid	NA	NA	NA	NA	6.8% (3/44)	NA	67% (6/9)
21	Volk 2016	Peru and Brazil	QE	103	Finger-prick	NA	15.5% (16 /103)	44.7% (46/103)	14.6% (15/103)	1.9% (2/103)	100% (2/2)	67% (6/9)
22	Han 2014	China	CS	1342	NA	NA	54.6% (434/795)	30.7% (245/797)	15.5% (206/1342)	6.1% (49/805)	NA	100% (8/8)
23	Chavez 2016	USA	QE	511	Oral-fluid and finger-prick	NA	NA	NA	NA	2.2% (11/511)	NA	67%(6/9)

NA, not available; CS, cross-sectional;QE, quasi-experimental;MM, mixed method (a survey and expert focus groups)

### Quality assessment

The methodological quality of the included studies was assessed using JBI-MAStARI standard appraisal tools[23] (S1 Doc), including tools for randomized controlled trials (S1 Table), cross-sectional studies (S2 Table), and quasi-experimental studies (S3 Table). A mixed-method designed study [24] was evaluated using the cross-sectional study checklist of JBI-MAStARI, as data were only extracted from the cross-sectional phase. A conference abstract [25] was evaluated using the Randomized Controlled Trials of the JBI-MAStARI appraisal checklist. After assessment of the quality by the two independent reviewers, disagreements were resolved through discussion. The risk of bias was evaluated according to Agency for Healthcare Research and Quality (AHRQ) methods[26] (S4 Table).

### Statistical analysis

Review Manager 5.3 was used to summarize the results. A meta-analysis was conducted on the efficacy of HIVST on improving HIV test frequency. Mean difference (MD) was applied as summary measures, with 95% confidence intervals (95% CI) reported. Mean Difference was calculated for HIV test frequency. If the pooled MD was higher than 0, it indicates an increase in the test frequency in the HIV self-testing group as compared to the standard-of-care group.

For the remaining meta-analyses of single proportion, the incidence rate P and the value of standard error were calculated with formulas: P = ln(x/(n-x)) and SE(P) = (1/x+1/(n-x))^1/2^, respectively. In the two formulas, x indicated the number of events, and n indicated the total number of objects observed. Odds ratio (OR) was calculated using the software of Review Manager 5.3, with 95% CI reported. The pooled percentage and 95% CI were calculated using formulas: P_t_ = OR/(1+OR), the lower limit of 95% CI: LL = LLOR/(1+LLOR) and the upper limit of 95% CI: UL = ULOR/(1+ULOR). In the three formulas, P_t_ indicated the pooled proportion, LLOR indicated the lower limit of OR, and ULOR indicated the upper limit of OR.

I-squared (I^2^) values were used to assess the impact of study heterogeneity. According to the Cochrane review guidelines, if I^2^ was less than 50%, the inverse variance fixed-effects model was chosen; otherwise, the DerSimonian-Laird random-effects model was used when clinical heterogeneity was big. If the number of included studies was more than 10, a funnel plot was conducted to assess publication bias [27,28]. For consistency, all figures were labeled “exp (HIVST)” vs “control (standard care),” even when studies were not strictly experimental.

## Results

A total of 1694 records were retrieved, 1661 from database searching, 26 from conference abstracts, and 7 from bibliography search. After duplicates were removed, 1321 records were identified. Records (n = 1088) were excluded if review of the title or abstract revealed that the topic was unrelated to HIV self-testing. The eligibility of the remaining 232 records was assessed by reviewing full-text articles (if available), and finally 23 studies were selected (21 full-text studies and 2 abstracts). Reasons for exclusion of the 209 studies are elaborated in Fig 1.

The 23 eligible studies were conducted from 2009 to 2015 and results published from 2012 to 2017 (including 4 in 2017, 9 in 2016, and 5 in 2015). Twenty-one studies were published in English and two in Chinese. Geographically, ten studies were conducted in the USA, eight in China, three in the United Kingdom, one in France, and one in Peru and Brazil. In other words, nine studies (39.1%, n = 9/23) were conducted in resource-constrained country settings [15,17,21,29–34] and 14 (60.9%, n = 14/23) in high-income countries [16,24,25,35–45]. The total sample size varied from 37 to 5696. The minimum age of participants in selected studies was 16 years old. An oral fluid-based test was the main testing method in the majority of studies (56.5%, n = 13/23), while a finger-prick based test alone was reported only in two studies; five studies reported using both testing methods, and four did not specify the product kits (Table 2). Only two studies were randomized controlled trials [25,35]; the majority were quasi-experimental (60.9%, n = 14/23) [21,30–34,36,38–40,42–45], while six were surveys (26.1%, n = 6/23)[15–17,29,37,41], and one was a mixed methods design (4.5%, n = 1/22) (survey and expert focus group) [23].

Reported outcome variables included frequency of HIV testing (3 studies), and the proportion of first time testers (12 studies), non-recent testers (12 studies), married MSM (7 studies), people who had HIV positive tests (21 studies), and linkage to care (14 studies) (Table 2). The quality of each study is evaluated below in detail in S2–S4 Tables. The quality appraisal scores were 100% in six studies (26.1%, n = 6/23) [29–31,35,36], 88% in two studies (8.7%, n = 2/23) [16,37], 67% in nine studies (43.5%, n = 10/23) [15,17,24,32,34,38,40,43–45], 56% in four studies (17.4%, n = 4/23) [33,39,41,42] and 46% in one study (4.3%, n = 1/23) [25] (S1–S3 Tables).

### The efficacy of HIVST to increase HIV test frequency

**Fig 2:**
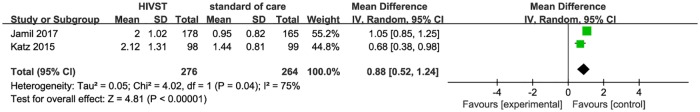
Meta-analysis on mean number of HIV tests for MSM in a 6-month period.

Only two RCT studies evaluated the efficacy of HIVST to improve HIV test frequency among MSM. One was carried out in Australia [35], and the other was in the USA [25]. The pooled study results indicated that MSM who were provided with HIVST kits as a supplement to site-based testing services had nearly one more test in a 6-month period than those who were provided with only site-based testing services (mean difference = 0.88; 95% CI 0.52, 1.24) (see Fig 2). In China, a nationwide online survey showed that 23% (n = 77/341) of those who ever took HIVST self-reported increasing HIV test frequency either by HIVST or site-based testing after first time using self-test kits[29].

In addition, the study in Australia also analyzed the testing frequency for delayed testers (those who had not been tested in the last 2 years or had never been tested before), and the results showed that MSM provided with HIVST as a supplementary option had 3.95 times higher test frequency than those men provided with only standard of care (Rate Ratio = 3.95; 95% CI 2.30, 6.78, P<0.0001)[35].

### The proportion of first time testers, non-recent testers, ever or currently married MSM and HIV positive MSM

The included studies, pooled proportions and ranges, ratios of the proportion in resource-limited countries/the proportion for all included studies for the four outcome variable of first time testers, non-recent testers, ever or currently married MSM and HIV positive MSM were listed in Table 3.

**Table 3:** The proportion of first-time testers, non-recent testers, married MSM, and HIV-positive MSM.

Outcomes	The included studies (n, ref.No)			The pooled proportion			Range	Ratio
	AIS	HIC	RLC	AIS	HIC	RLC		
First-time testers	12[15,17,21,24,29,30,32,34,37,40,41,43]	6(50%, n = 6/12) [24,30,37,40,41,43]	6 (50%, n = 6/12) [15,17,21,29,32,34]	18.7%(95% CI: 9.9–32.4%) (Fig 3)	9.9% (95% CI: 7.4–13.8%) (Fig 4)	32.9%(95% CI: 21.3–47.6%) (Fig 5)	From 5.8%, (n = 62/1071) [40] to 58.7%, (n = 200/341) [29]	RLC/HIC: 3.32 RLC/AIS: 1.76
Non-recent testers	12 [15,21,25,30,34–37,39,41,43]	8 (66.7%, n = 8/12) [30,35–37,39–41,43]	4 (33.3%, n = 4/12) [15,21,25,34]	32.9% (95% CI: 28.1–38.3%) (Fig 6)	33.8% (95%CI: 27.5–40.8%) (Fig 7)	31.5% (95% CI: 24.8–39.4%) (Fig 8)	From 15.7% (n = 57/362) [35] to 59% (n = 117/143) [29]	RLC/HIC: 0.93 RLC/AIS:0.95
Ever or currently married MSM	7 [15,17,21,30,31,34]	6 (85.7%, n = 1/7) [15,17,21,31,32,34]	1 (14.3%, n = 1/7) [30]	16.7% (95% CI: 14.5–19.4%) (Fig 9)	NA	NA	From (8.1%, n = 16/178) [21] to (21.5%, n = 43/200) [31]	NA
HIV positive men	21[15,16,21,24,29–45]	14 (66.7%, n = 14/21), [16,24,30,35–45]	7 (33.3%, n = 7/21) [15,21,29,31–34]	3.8% (95% CI: 2–5.7%) (Fig 10)	2.9% (95% CI: 2–5%) (Fig 11)	6.5% (95% CI: 3.8%-12.3%) (Fig 12)	From 0.5% (n = 2/384)[33] to 23% (n = 46/200) [29]	RLC/HIC:2.24 RLC/AIS:1.71

AIS: for all included studies; HIC: High-income countries: RLC: Resource-limited countries; NA: not available. RLC/AIS: The proportion in resource-limited countries/the proportion for all included studies. RLC/HIC: The proportion in resource-limited countries/the proportion in high-income countries

**Fig 3:**
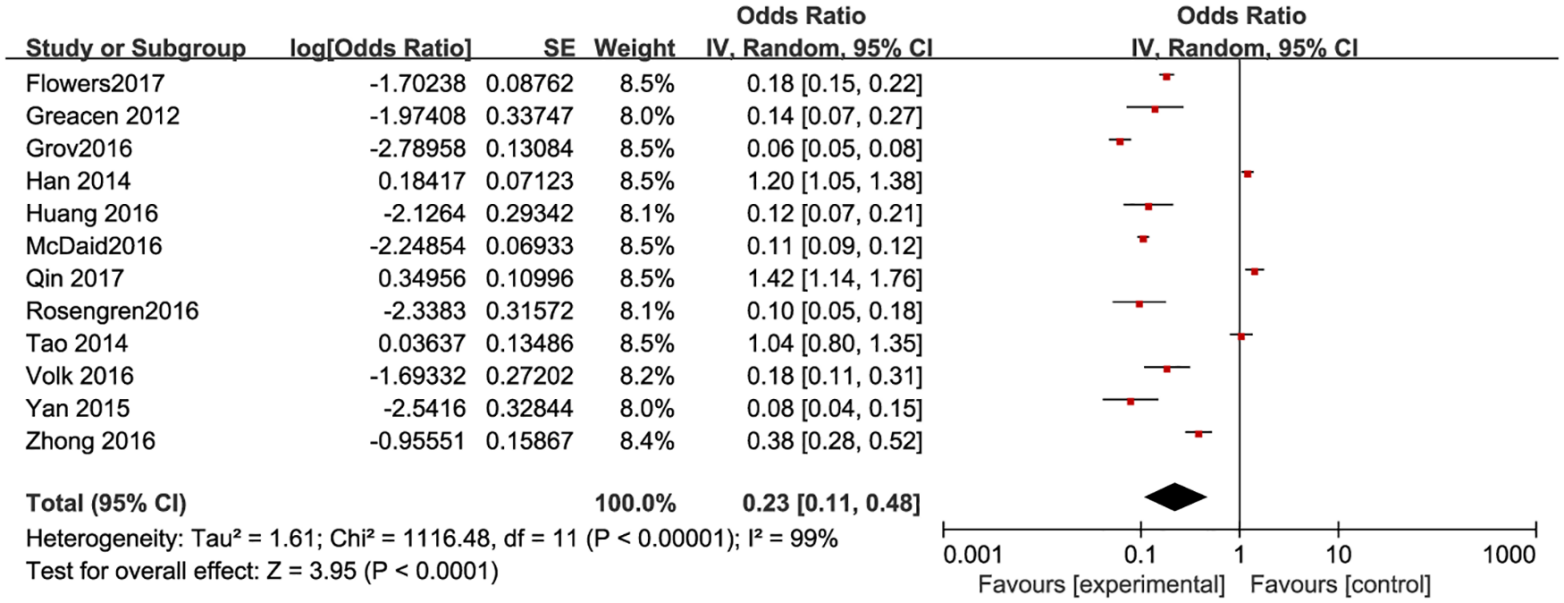
Meta-analysis of the proportion of first-time testers among MSM ever taking HIVST. Without comparison, this figure just presented OR value of pooled single proportion.

**Fig 4:**
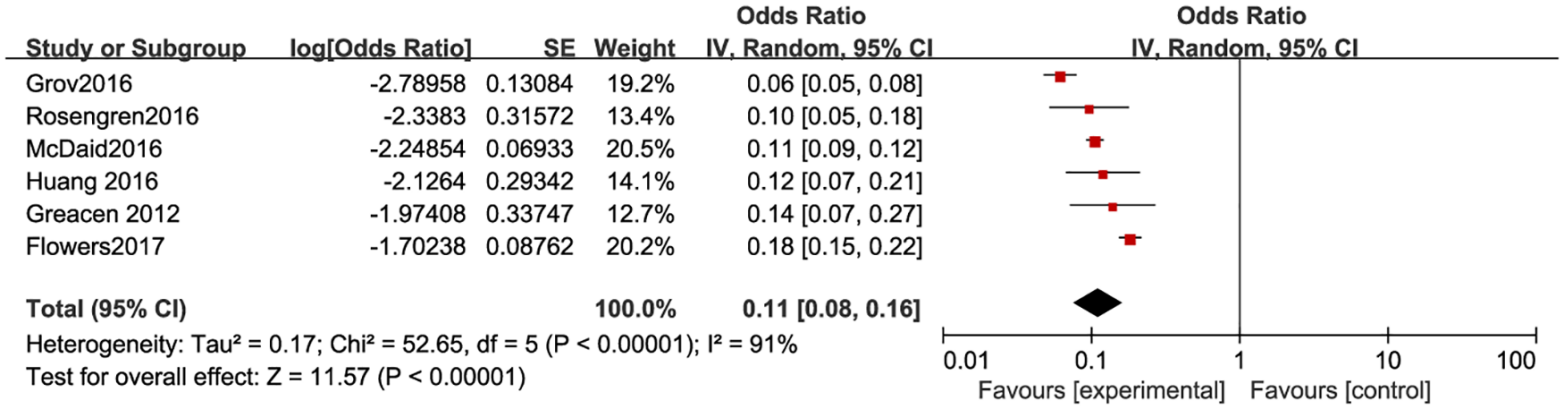
Meta-analysis of the proportion of first-time testers in high-income countries. Without comparison, this figure just presented OR value of pooled single proportion.

**Fig 5:**
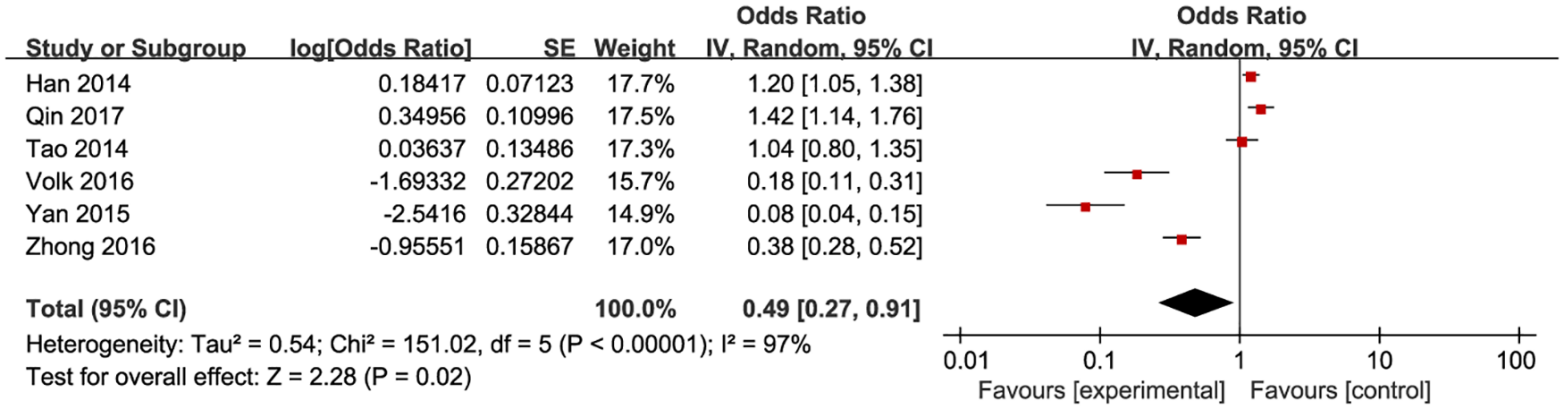
Meta-analysis of the proportion of first-time testers in resource constrained countries. Without comparison, this figure just presented OR value of pooled single proportion.

The pooled proportion of the first time testers among those taking HIVST was 18.7% (95% CI: 9.9–32.4%) for the 12 included studies (Fig 3). The pooled estimated proportion of first-time testers was 9.9% (95% CI: 7.4–13.8%) in high-income countries (Fig 4), and 32.9% (95% CI: 21.3–47.6%) (Fig 5) in resource-limited countries, which was 3.32 times higher than the percentage for high-income countries and 1.76 times higher than the total combined percentage.

**Fig 6:**
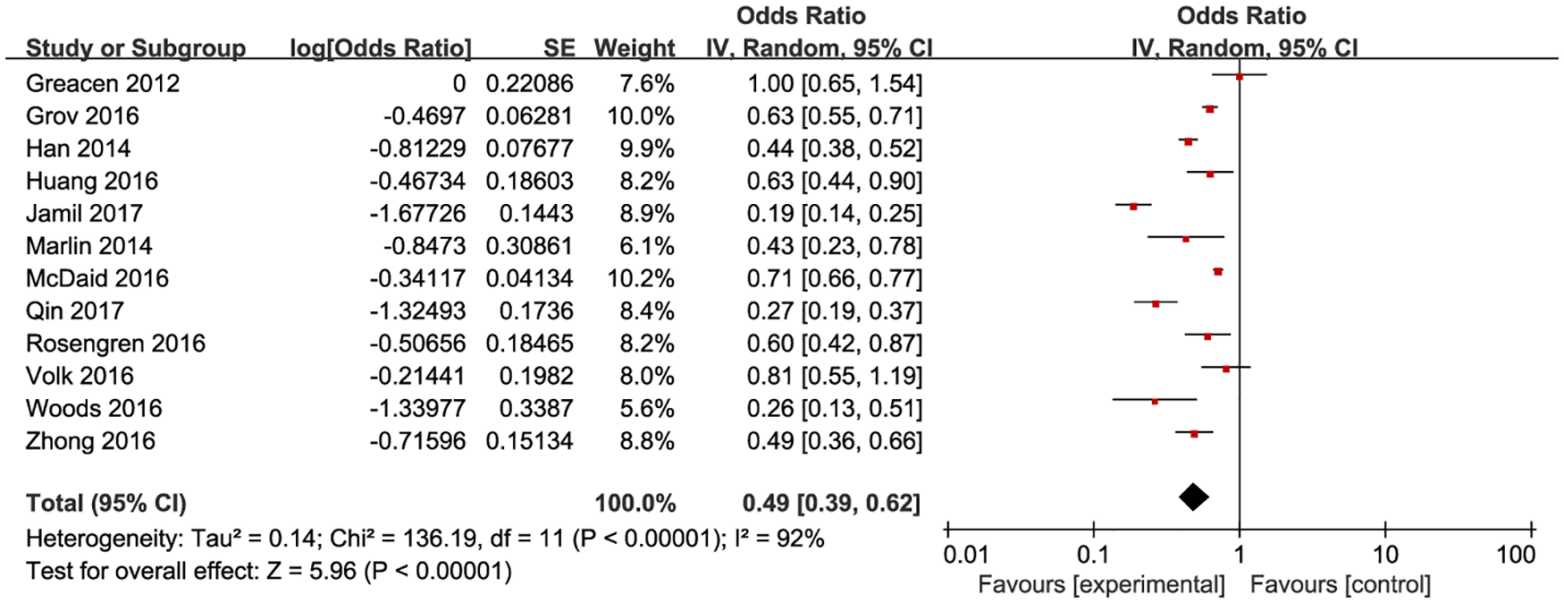
Meta-analysis of the proportion of non-recent testers among MSM. Without comparison, this figure just presented OR value of pooled single proportion.

**Fig 7:**
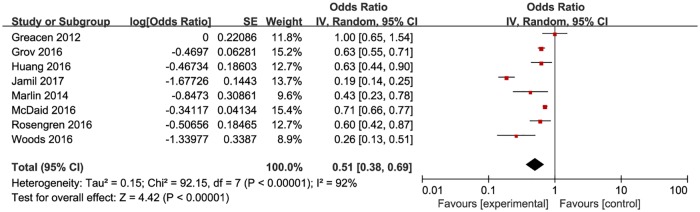
Meta-analysis of the proportion of non-recent testers in high-income countries. Without comparison, this figure just presented OR value of pooled single proportion.

**Fig 8:**
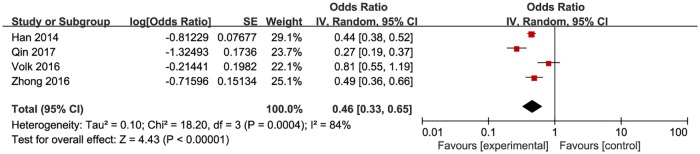
Meta-analysis of the proportion of non-recent testers in resource constrained countries. Without comparison, this figure just presented OR value of pooled single proportion.

The proportion of non-recent testers accounting for MSM who ever took HIVST varied from 15.7% (n = 57/362) in Australia [35] to 59% (n = 117/143) in China [29]. The pooled percentage of non-recent testers was 32.9% (95% CI: 28.1–38.3%) (Fig 6) overall, 33.8% (95% CI: 27.5–40.8%) in high-income countries (Fig 7), and 31.5% (95% CI: 24.8–39.4%) in resource-constrained country settings (Fig 8).

**Fig 9:**
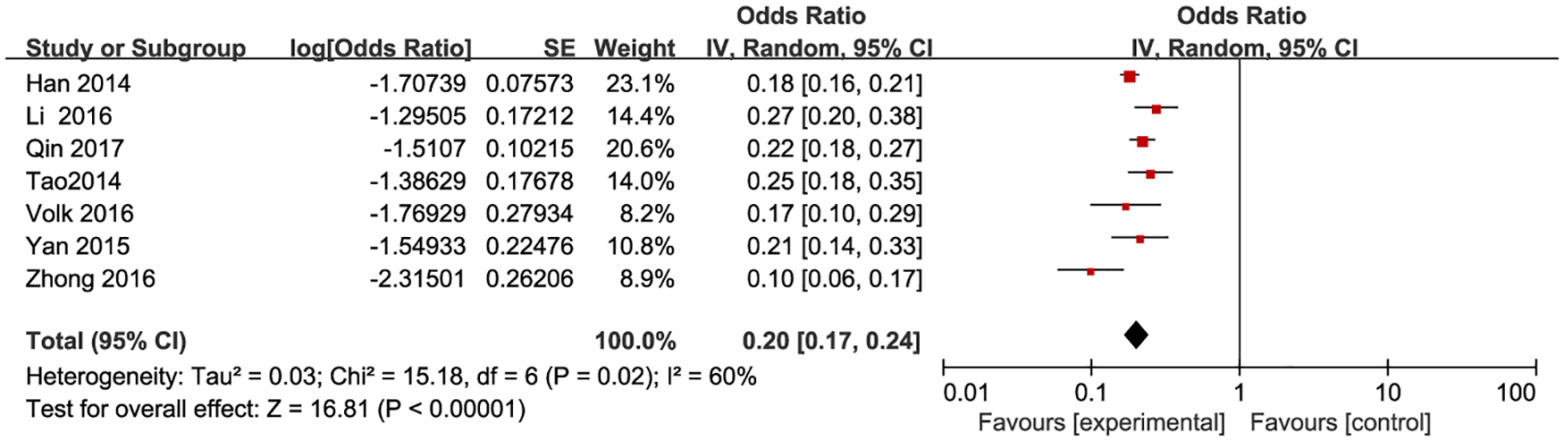
Meta-analysis of the proportion of ever or currently married MSM. Without comparison, this figure just presented OR value of pooled single proportion.

The pooled percentage of ever or currently married MSM was 16.7% (95% CI: 14.5–19.4%) (Fig 9). The lowest and highest rates of ever or currently married MSM were (8.1%, n = 16/178) [21] in Guangzhou province of China and (21.5%, n = 43/200) [31] in Yunnan province of China, respectively.

**Fig 10:**
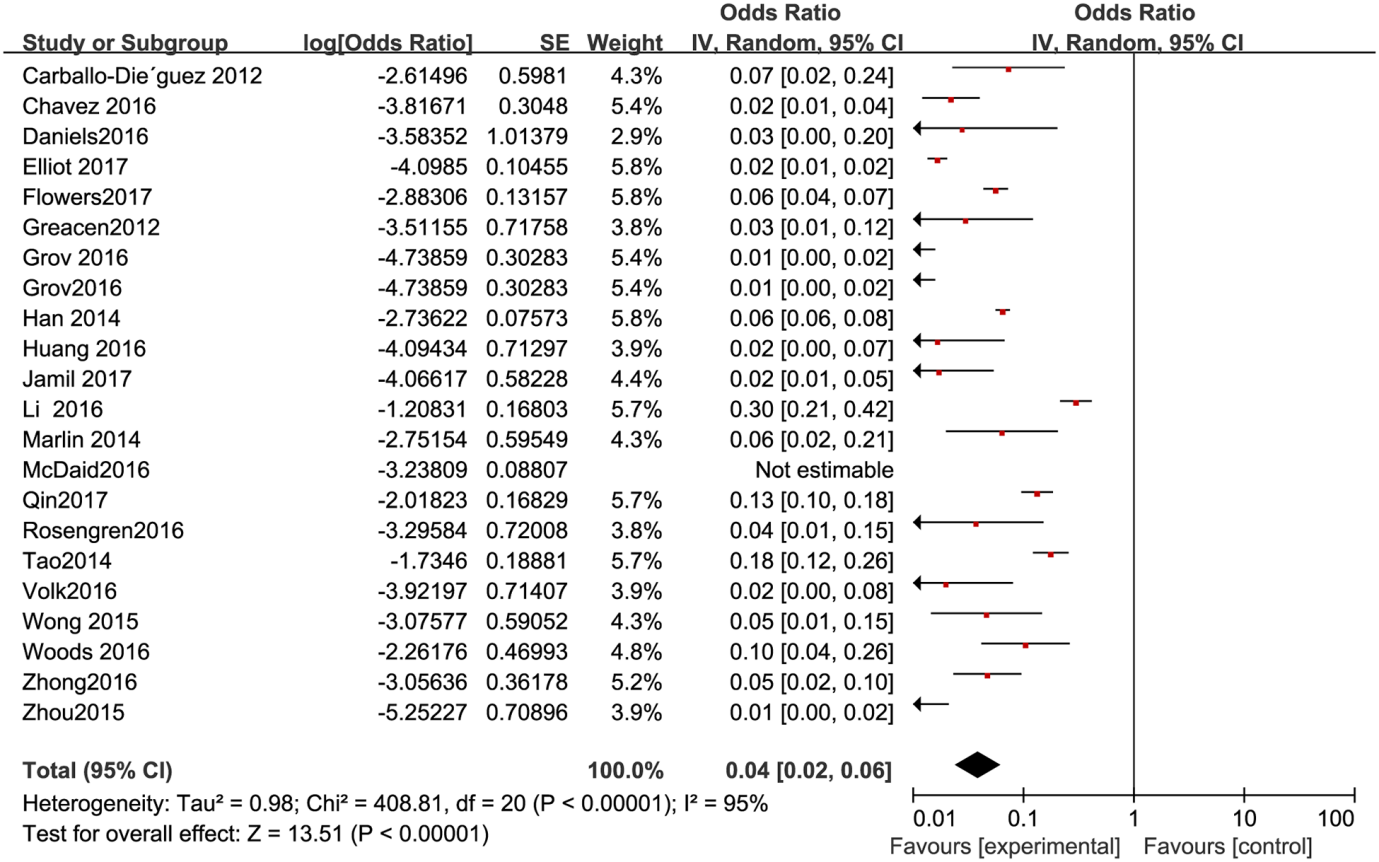
Meta-analysis of the proportion of HIV-positive men among MSM. Without comparison, this figure just presented OR value of pooled single proportion.

**Fig 11:**
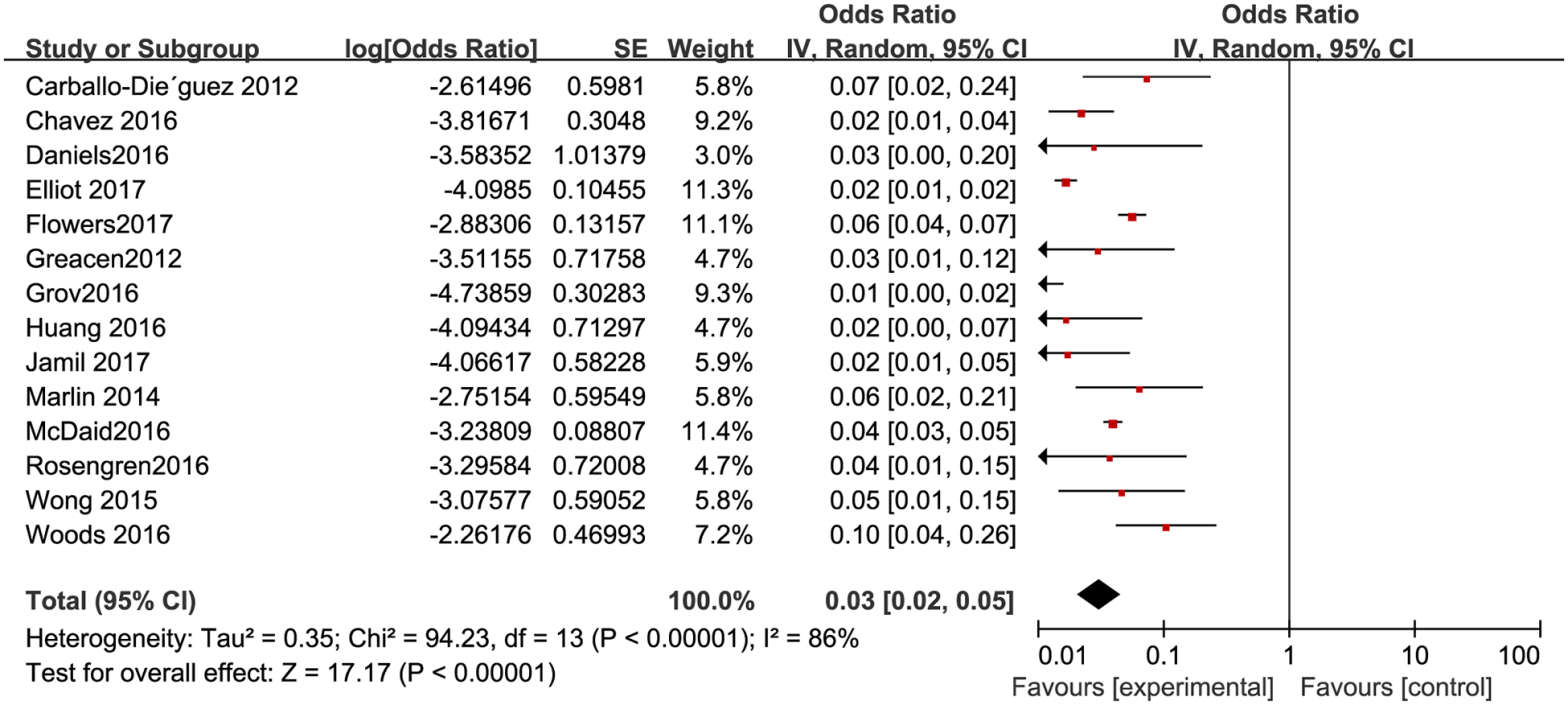
Meta-analysis of the proportion of HIV-positive men in high-income countries. Without comparison, this figure just presented OR value of pooled single proportion.

**Fig 12:**
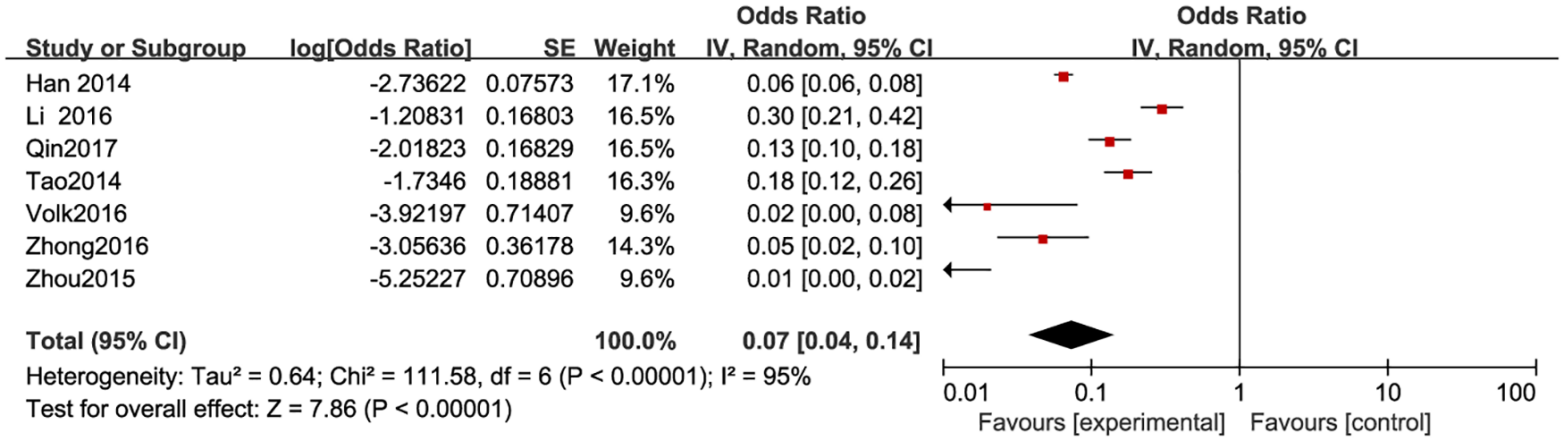
Meta-analysis of the proportion of HIV-positive men in resource-limited countries. Without comparison, this figure just presented OR value of pooled single proportion.

The proportion of HIV positive individuals varied across studies, with the lowest proportion of 0.5% (n = 2/384) in Beijing [33] and the highest proportion of 23% (n = 46/200) in Yunnan, China [29]. The pooled proportion was 3.8% (95% CI: 2–5.7%) (Fig 10). The pooled proportion of HIV positive individuals was 2.9% (95% CI: 2–5%) in high-income countries (Fig 11), and 6.5% (95% CI: 3.8%-12.3%) (Fig 12) in resource-constrained countries(1.71 times higher than the total pooled proportion and 2.24 times than in the high-income countries).

### Linkage-to-care rate

Fourteen studies evaluated the linkage-to-care rate for HIV-positive individuals [16,21,29,30,32–40,43]. Eight studies were conducted in high-income countries [16,35–40,43], while six [21,29,30,32–34] were in resource-limited countries. HIV-positive individuals were all linked to care in nine studies [16,21,30,32,34–36,40,43]. In the remaining five studies, the percentages of linkage to care varied, with 31.3% (n = 5/16) [33] and 77.5% (n = 31/40) in China [29], 66.7%(n = 2/3) in France [37], 80% (n = 4/5) in the USA [39], 88% (n = 82/93) in the United Kingdom[38].

## Discussion

Although HIVST was first introduced by Joint United Nations Programs on HIV/AIDS in 2000, it has not been widely implemented globally [46]. One of the main reasons is due to the lack of scientific evidences on the effectiveness of HIVST to promote HIV testing program. Potential harms of HIVST without supervision and disengagement in HIV care for self-testers with positive results exacerbate the concerns [21]. The 23 articles/abstracts we retrieved in this meta-analysis were all published in the last 5 years. Few studies globally produced rigorous evidence on evaluation of HIVST to improve HIV testing for MSM, with only two RCT-based studies. Most of the studies were carried out in United States of America (10 studies) and China (8 studies), possibly because we restricted the languish only to English and Chinese. However, China is currently on its way to enlarge HIV testing program by integrating HIVST approach, which is illustrated by China’s “The 13th Five-year” plan on HIV/AIDS prevention and control (2017), which put an emphasis on promoting HIVST among MSM and their sexual partners[47].

Globally, HIV test frequency is low among MSM, especially in resource limited countries. The average number of site-based HIV testing was 0.47 test every 6 months for MSM in Australia[48], while in China, the average number of lifetime HIV tests was only 1–2 [6].Our meta-analysis showed that HIVST could improve HIV test frequency for MSM by one more test every 6 months, which has the substantial potential to assist the traditional HIV testing service to meet the guideline recommendation that MSM with high risk behaviors (unprotected anal sex, multiple sex partners, use of recreational drugs, etc.) need a HIV test every 3–6 months[49,50].As a supplementary testing service, HIVST could especially increase the test frequency 3.95 times for those delayed testers, who usually complained of the long distance transportation to the site-based services, long waiting time for the results, and the risk of being stigmatized by people working in the site-based service center[51].

Oral-fluid based HIVST kits have been approved by authority in the U.S.A.,U.K and France [35]. However, it has not received approval from the regulators in Hong Kong, China, Australia and Brazil[35]. Oral-fluid kits are less painful but more expensive than finger-prick kits which might be more acceptable in resource limited countries. In our meta-analysis, there were RCTs to explore the effectiveness of the oral-fluid kits to improve HIV testing frequency, but none were using finger-prick kits. Thus, more RCT studies in different cultural and economic settings are needed to provide more evidence for the efficacy of finger-prick kits to increase HIV testing frequency.

Having the non-testers and delayed testers of MSM get HIV tests is the main goal of expanding HIV testing among the high risk population. But is the HIVST superior than site-based testing services to reach those high risk population? Based on the current evidence, 18.7% of HIV self testers were first time testers, and 32.9% were non-recent testers, similar with the proportion of site-based HIV testing services among these two outcomes, 11.9%-34.9% for the first time testers among MSM [52,53] and 26.6%-33.3% for the non-recent testers [54–56]. Thus, HIVST has equivalent potential with site-based HIV testing to reach those at high risk who are not accessing traditional HIV testing services. However, the capacity of HIVST to reach that high-risk population is different depending on the social economic status in the study settings. Our meta-analysis showed that HIVST could reach 3.32 times more first time testers in resource-limited countries (32.9%) than in high-income countries (9.9%). The rationale is that free providing of HIVST kits during the studies became an incentive for economically vulnerable MSM. This is a significant evidence implying that free or low cost of HIVST services could be much more effective to enlarge HIV testing in resource limited countries than high income countries. Because the cost of the self-testing kits has been proved to be a big barrier for people to use it [21,57,58]. Thus, social economic status is an important factor when implementing HIVST services.

Unlike western developed countries, a significant proportion of MSM in developing countries married or would marry with female, for example, currently 17.0% (95% CI: 15.1%–19.1%) of MSM were married with women in China[59], and 30%-40% of MSM were married with women in India[60,61]. With comparable HIV burdens to unmarried MSM [62], only 7.1% of HIV positive married MSM ever took HIV testing last year [62]. Married MSM were assumed to have more barriers to having an HIV test [63]. However, no summarized evidence showed the effectiveness of HIVST to reach this hidden subgroup of men. Our meta-analysis showed married MSM accounted for 16.7% of MSM who had ever taken HIVST. This was a comparable proportion to that of MSM who had taken a site-based HIV test (around 16%-17%) [44, 47]. Therefore, HIVST doesn’t have a superior capacity to reach the hidden population of married MSM. Recommendation for future research may include qualitative study to examine barriers and facilitators of the implementation of HIVST for married MSM. Besides, HIVST could be provided as a complementary testing approach to site-based testing services, in order to better reach this hidden population, and prevent HIV transmission from MSM to their female partners.

According to Global AIDS Response Progress Reporting data from 96 countries, the median HIV prevalence among MSM is 3.7% [64]. The proportion of HIV positive MSM in China was 6%-8% reported by UNAIDS [50].Our meta-analysis results revealed the similar proportions (3.8% in total, 6.5% in resource-limited countries, and 2.9% in high-income countries).Thus, HIVST has a similar potential to reach individuals living with HIV compared with the site-based HIV testing approach. The current evidence also showed that HIVST did not reduce the linkage to care rate (most HIV-positive individuals were linked to care in the retrieved studies). However, there is still a gap to meet the 90-90-90 target goal. Strategies to facilitate the linkage to care for self-tested HIV positive MSM are needed to maximize the benefits of HIVST.

## Limitations

This meta-analysis has several limitations. First, the evidence included in the data synthesis on the efficacy of HIVST to improve HIV test frequency was not as strong as expected, since there were only two RCT-designed studies, and both were implemented in high-income countries. Lack of data from resource-limited countries constrains our ability to examine the efficacy of HIVST on improving the HIV test frequency in diverse country settings. Second, selection bias cannot be ruled out because languages were limited to English and Chinese. Third, information bias also likely exists, because some outcomes were evaluated by the participants’ self-reporting. The risk of bias within the studies was assessed using Agency for Healthcare Research and Quality (AHRQ) methods, but some assessment items were not applicable due to the type of study design which limits our ability to make a graph for the risk of bias. Fourth, the heterogeneity across included studies was high in this meta-analysis. The main reasons might be that the outcomes lacked standard definitions across the studies (such as the "non-recent testers") and samples were recruited from diverse settings.

## Conclusions and implications

Our meta-analysis indicated that as a complementary approach for HIV site-based testing services, HIVST has potential to improve test frequency and has the equivalent capacity to reach the first-time, delayed, married and HIV-infected testers among MSM, and could effectively link HIV-infected MSM to medical care. Thus HIVST could potentially contribute to achieving WHO’s 90-90-90 target goal, and specifically the first target of diagnosing 90% of all people with HIV. However, because of the limited studies on HIVST, we do not know which self-testing approach (oral-fluid based, or finger-prick based) is more acceptable and effective in term of improving HIV testing for MSM. More rigorous study designs are needed to explore the specific self testing approach on improving HIV testing for MSM in certain social economic settings.

## Supporting information

S1 Doc JBI critical appraisal tools. (DOCX)

S1 Table Assessment of methodological quality of randomized controlled trials(n = 2). (DOCX)

S2 Table Assessment of methodological quality of cross sectional studies (n = 6). (DOCX)

S3 Table Assessment of methodological quality of quasi-experimental studies (n = 14). (DOCX)

S4 Table Risk of bias assessment within the studies (n = 23). (DOCX)

S1 PRISMA Checklist (DOC)
