# Supplementary material for: Can self-testing increase HIV testing among men who have sex with men: A systematic review and meta-analysis
Source: PLoS One. 2017 Nov 30;12(11):e0188890. doi: 10.1371/journal.pone.0188890 (PMC5708824; doi:10.1371/journal.pone.0188890)
Supplement: S1 Doc — (DOCX) [file pone.0188890.s001.docx]

**JBI Critical Appraisal Checklist for Analytical Cross Sectional Studies**

Reviewer Date

Author Year Record Number

|  | Yes | No | Unclear | Not applicable |
| --- | --- | --- | --- | --- |
| 1. Were the criteria for inclusion in the sample clearly defined? | □ | □ | □ | □ |
| 1. Were the study subjects and the setting described in detail? | □ | □ | □ | □ |
| 1. Was the exposure measured in a valid and reliable way? | □ | □ | □ | □ |
| 1. Were objective, standard criteria used for measurement of the condition? | □ | □ | □ | □ |
| 1. Were confounding factors identified? | □ | □ | □ | □ |
| 1. Were strategies to deal with confounding factors stated? | □ | □ | □ | □ |
| 1. Were the outcomes measured in a valid and reliable way? | □ | □ | □ | □ |
| 1. Was appropriate statistical analysis used? | □ | □ | □ | □ |

Overall appraisal: Include □ Exclude □ Seek further info □

Comments (Including reason for exclusion)

**JBI Critical Appraisal Checklist for Quasi-Experimental Studies (non-randomized experimental studies)**

Reviewer Date

Author Year Record Number

|  | Yes | No | Unclear | Not applicable |
| --- | --- | --- | --- | --- |
| 1. Is it clear in the study what is the ‘cause’ and what is the ‘effect’ (i.e. there is no confusion about which variable comes first)? | □ | □ | □ | □ |
| 2. Were the participants included in any comparisons similar? | □ | □ | □ | □ |
| 3. Were the participants included in any comparisons receiving similar treatment/care, other than the exposure or intervention of interest? | □ | □ | □ | □ |
| 4. Was there a control group? | □ | □ | □ | □ |
| 5. Were there multiple measurements of the outcome both pre and post the intervention/exposure? | □ | □ | □ | □ |
| 6. Was follow-up complete, and if not, was follow-up adequately reported and strategies to deal with loss to follow-up employed? | □ | □ | □ | □ |
| 7. Were the outcomes of participants included in any comparisons measured in the same way? | □ | □ | □ | □ |
| 8. Were outcomes measured in a reliable way? | □ | □ | □ | □ |
| 1. Was appropriate statistical analysis used? | □ | □ | □ | □ |

Overall appraisal: Include □ Exclude □ Seek further info □

Comments (Including reason for exclusion)

**JBI Critical Appraisal Checklist for Randomized Controlled Trials**

Reviewer Date

Author Year Record Number

|  | Yes | No | Unclear | NA |
| --- | --- | --- | --- | --- |
| 1. Was true randomization used for assignment of participants to treatment groups? | □ | □ | □ | □ |
| 2. Was allocation to treatment groups concealed? | □ | □ | □ | □ |
| 3. Were treatment groups similar at the baseline? | □ | □ | □ | □ |
| 4. Were participants blind to treatment assignment? | □ | □ | □ | □ |
| 5. Were those delivering treatment blind to treatment assignment? | □ | □ | □ | □ |
| 6. Were outcomes assessors blind to treatment assignment? | □ | □ | □ | □ |
| 7. Were treatments groups treated identically other than the intervention of interest? | □ | □ | □ | □ |
| 8. Was follow-up complete, and if not, were strategies to address incomplete follow-up utilized? | □ | □ | □ | □ |
| 9. Were participants analysed in the groups to which they were randomized? | □ | □ | □ | □ |
| 10. Were outcomes measured in the same way for treatment groups? | □ | □ | □ | □ |
| 11. Were outcomes measured in a reliable way? | □ | □ | □ | □ |
| 12. Was appropriate statistical analysis used? | □ | □ | □ | □ |
| 13. Was the trial design appropriate, and any deviations from the standard RCT design (individual randomization, parallel groups) accounted for in the conduct and analysis of the trial? | □ | □ | □ | □ |

Overall appraisal: Include □ Exclude □ Seek further info □

Comments (Including reason for exclusion)
