# Supplementary material for: Can self-testing increase HIV testing among men who have sex with men: A systematic review and meta-analysis
Source: PLoS One. 2017 Nov 30;12(11):e0188890. doi: 10.1371/journal.pone.0188890 (PMC5708824; doi:10.1371/journal.pone.0188890)
Supplement: S1 Table — (DOCX) [file pone.0188890.s002.docx]

**S1 Table. Assessment of methodological quality of Randomized Controlled Trials(n=2)**

| Authors | Q1 | Q2 | Q3 | Q4 | Q5 | Q6 | Q7 | Q8 | Q9 | Q10 | Q11 | Q12 | Q13 | % |
| --- | --- | --- | --- | --- | --- | --- | --- | --- | --- | --- | --- | --- | --- | --- |
| Jamil et al | Y | Y | Y | Y | Y | Y | Y | Y | Y | Y | Y | Y | Y | 100 |
| Katz et al | Y | NC | NC | NC | NC | NC | NC | Y | NC | Y | Y | Y | Y | 46 |

Q= Question ;Y=Yes; N= No; ; NC = Nuclear; NA= Not applicable
