# Supplementary material for: Can self-testing increase HIV testing among men who have sex with men: A systematic review and meta-analysis
Source: PLoS One. 2017 Nov 30;12(11):e0188890. doi: 10.1371/journal.pone.0188890 (PMC5708824; doi:10.1371/journal.pone.0188890)
Supplement: S2 Table — (DOCX) [file pone.0188890.s003.docx]

**S2Table . Assessment of methodological quality of cross sectional studies(n=6)**

| Authors | Q1 | Q2 | Q3 | Q4 | Q5 | Q6 | Q7 | Q8 | % |
| --- | --- | --- | --- | --- | --- | --- | --- | --- | --- |
| Qin et al | Y | Y | Y | Y | Y | Y | Y | Y | 100 |
| Wong et al | Y | Y | Y | Y | Y | Y | Y | Y | 100 |
| Greacen et al | Y | Y | Y | Y | Y | Y | Y | Y | 100 |
| Yan et al | Y | Y | Y | Y | Y | Y | Y | Y | 100 |
| McDaid et al | N | Y | Y | Y | Y | Y | Y | Y | 88 |
| Folwers et al | N | Y | Y | Y | Y | Y | Y | Y | 88 |
| Han et al | Y | Y | Y | Y | Y | Y | Y | Y | 100 |

Q= Question ; Y=Yes; N= No; NA= Not applicable
