# Supplementary material for: Can self-testing increase HIV testing among men who have sex with men: A systematic review and meta-analysis
Source: PLoS One. 2017 Nov 30;12(11):e0188890. doi: 10.1371/journal.pone.0188890 (PMC5708824; doi:10.1371/journal.pone.0188890)
Supplement: S3 Table — (DOCX) [file pone.0188890.s004.docx]

**S3 Table. Assessment of methodological quality of Quasi-Experimental studies(n=14)**

| Authors | Q1 | Q2 | Q3 | Q4 | Q5 | Q6 | Q7 | Q8 | Q9 | % |
| --- | --- | --- | --- | --- | --- | --- | --- | --- | --- | --- |
| Marlin et al | Y | NA | Y | N | NA | Y | Y | Y | Y | 67% |
| Huang et al | Y | NA | Y | N | NA | Y | Y | Y | Y | 67% |
| Li et al | Y | NA | Y | N | NA | Y | Y | Y | Y | 67% |
| Zhong et al | Y | NA | N | N | NA | Y | Y | Y | Y | 56% |
| Tao et al | Y | NA | N | N | NA | Y | Y | Y | Y | 56% |
| Elliot et al | Y | NA | Y | N | NA | Y | Y | Y | Y | 67% |
| Woods et al | Y | NA | N | N | NA | Y | Y | Y | Y | 56% |
| Zhou et al | Y | NA | N | N | NA | Y | Y | Y | Y | 56% |
| Grov et al | Y | NA | Y | N | NA | Y | Y | Y | Y | 67% |
| Daniels et al | Y | NA | Y | N | NA | Y | Y | Y | Y | 67% |
| Rosengren et al | Y | NA | Y | N | NA | Y | Y | Y | Y | 67% |
| Carballo-D et al | Y | NA | Y | N | NA | Y | Y | Y | Y | 67% |
| Volk et al | Y | NA | Y | N | NA | Y | Y | Y | Y | 67% |
| Chavez et al | Y | NA | Y | N | NA | Y | Y | Y | Y | 67% |

Q= Question ;Y=Yes; N= No; ; NA= Not applicable
