# Supplementary material for: Can self-testing increase HIV testing among men who have sex with men: A systematic review and meta-analysis
Source: PLoS One. 2017 Nov 30;12(11):e0188890. doi: 10.1371/journal.pone.0188890 (PMC5708824; doi:10.1371/journal.pone.0188890)
Supplement: S4 Table — (DOCX) [file pone.0188890.s005.docx]

**S4 Table. Risk of Bias Assessment within the studies (n=23)**

| Study | Random Sequence Generation (Selection bias) | Allocation Concealment (Selection bias) | Blinding of Participants and personnel (Performance bias) | Blinding of outcome Assessment (Detection bias) | Incomplete Outcome Data (attrition bias) | Selective reporting (Reporting bias) | Other |
| --- | --- | --- | --- | --- | --- | --- | --- |
| Jmail et al | Low risk | Low risk | Low risk | Low risk | Low risk | Low risk | Low risk |
| Katz et al | Low risk | Unclear risk | Unclear risk | Unclear risk | Unclear risk | Low risk | Low risk |
| Marlin et al | Unclear risk^a^ | Unclear risk^a^ | Unclear risk | Unclear risk | Low risk | Low risk | Low risk |
| Qin et al | Unclear risk^a^ | Unclear risk^a^ | Unclear risk | Unclear risk | Low risk | Low risk | Low risk |
| Huang et al | Unclear risk^a^ | Unclear risk^a^ | Unclear risk | Unclear risk | Low risk | Low risk | Low risk |
| Li et al | Unclear risk^a^ | Unclear risk^a^ | Unclear risk | Unclear risk | Low risk | Low risk | Low risk |
| Wong et al | Unclear risk^a^ | Unclear risk^a^ | Unclear risk | Unclear risk | Low risk | Low risk | Low risk |
| Greacen et al | Unclear risk^a^ | Unclear risk^a^ | Unclear risk | Unclear risk | Low risk | Low risk | Low risk |
| Zhong et al | Unclear risk^a^ | Unclear risk^a^ | Unclear risk | Low risk | Low risk | Low risk | Low risk |
| Tao et al | Unclear risk^a^ | Unclear risk^a^ | Unclear risk | Low risk | Low risk | Low risk | Low risk |
| Yan et al | Unclear risk^a^ | Unclear risk^a^ | Unclear risk | Low risk | Low risk | Low risk | Low risk |
| Elliot et al | Unclear risk^a^ | Unclear risk^a^ | Unclear risk | Low risk | Low risk | Low risk | Low risk |
| Woods et al | Unclear risk^a^ | Unclear risk^a^ | Unclear risk | Low risk | Low risk | Low risk | Low risk |
| Zhou et al | Unclear risk^a^ | Unclear risk^a^ | Unclear risk | Low risk | Low risk | Low risk | Low risk |
| Grov et al | Unclear risk^a^ | Unclear risk^a^ | Unclear risk | Low risk | Low risk | Low risk | Low risk |
| McDaid et al | Unclear risk^a^ | Unclear risk^a^ | Unclear risk | Low risk | Low risk | Low risk | Low risk |
| Daniels et al | Unclear risk^a^ | Unclear risk^a^ | Unclear risk | Low risk | Low risk | Low risk | Low risk |
| Flowers et al | Unclear risk^a^ | Unclear risk^a^ | Unclear risk | Low risk | Low risk | Low risk | Low risk |
| Rosengren et al | Unclear risk^a^ | Unclear risk^a^ | Unclear risk | Low risk | Low risk | Low risk | Low risk |
| Carballo-D et al | Unclear risk^a^ | Unclear risk^a^ | Unclear risk | Low risk | Low risk | Low risk | Low risk |
| Volk et al | Unclear risk^a^ | Unclear risk^a^ | Unclear risk | Low risk | Low risk | Low risk | Low risk |
| Han et al | Unclear risk^a^ | Unclear risk^a^ | Unclear risk | Unclear risk | Low risk | Low risk | Low risk |
| Chavez et al | Unclear risk^a^ | Unclear risk^a^ | Unclear risk | Low risk | Low risk | Low risk | Low risk |

a = Not applicable due to type of study design
